# An epigenetic breeding system in soybean for increased yield and stability

**DOI:** 10.1111/pbi.12919

**Published:** 2018-05-07

**Authors:** Sunil K. Kenchanmane Raju, Mon‐Ray Shao, Robersy Sanchez, Ying‐Zhi Xu, Ajay Sandhu, George Graef, Sally Mackenzie

**Affiliations:** ^1^ Department of Agronomy and Horticulture University of Nebraska‐Lincoln Lincoln NE USA; ^2^ Present address: Departments of Biology and Plant Science Pennsylvania State University University Park PA USA; ^3^ Present address: Syngenta Woodland CA USA

**Keywords:** GxE, transcriptome, heterosis, plant memory, RNA interference

## Abstract

Epigenetic variation has been associated with a wide range of adaptive phenotypes in plants, but there exist few direct means for exploiting this variation. RNAi suppression of the plant‐specific gene, *MutS HOMOLOG1* (*MSH1*), in multiple plant species produces a range of developmental changes accompanied by modulation of defence, phytohormone and abiotic stress response pathways along with methylome repatterning. This *msh1‐*conditioned developmental reprogramming is retained independent of transgene segregation, giving rise to transgene‐null ‘memory’ effects. An isogenic memory line crossed to wild type produces progeny families displaying increased variation in adaptive traits that respond to selection. This study investigates amenability of the *MSH1* system for inducing agronomically valuable epigenetic variation in soybean. We developed MSH1 epi‐populations by crossing with *msh1‐*acquired soybean memory lines. Derived soybean epi‐lines showed increase in variance for multiple yield‐related traits including pods per plant, seed weight and maturity time in both glasshouse and field trials. Selected epi‐F_2:4_ and epi‐F_2:5_ lines showed an increase in seed yield over wild type. By epi‐F_2:6,_ we observed a return of *MSH1*‐derived enhanced growth back to wild‐type levels. Epi‐populations also showed evidence of reduced epitype‐by‐environment (e × E) interaction, indicating higher yield stability. Transcript profiling of epi‐lines identified putative signatures of enhanced growth behaviour across generations. Genes related to cell cycle, abscisic acid biosynthesis and auxin response, particularly SMALL AUXIN UP RNAs (SAURs), were differentially expressed in epi‐F_2:4_ lines that showed increased yield when compared to epi‐F_2:6_. These data support the potential of *MSH1*‐derived epigenetic variation in plant breeding for enhanced yield and yield stability.

## Introduction

Plants respond to changing environments through phenotypic plasticity that derives from both genetic and epigenetic factors (Bossdorf *et al*., 2010; Kooke *et al*., 2015). Epigenetic variation can, to some extent, be monitored via cytosine DNA methylation repatterning (Becker *et al*., 2011; Schmitz *et al*., 2011) that can be transgenerationally heritable (Quadrana and Colot, 2016). Global changes in DNA methylation patterns in response to various environmental stresses have been reported in multiple plant species (Boyko *et al*., 2010; Karan *et al*., 2012; Wang *et al*., 2014). *Arabidopsis* epigenetic recombinant inbred lines (epiRILs), derived from crossing wild‐type *Col‐0* with *met1* or *ddm1* DNA methylation mutants, show segregation and heritability of novel methylation patterns together with phenotypic diversity (Johannes *et al*., 2009; Reinders *et al*., 2009; Roux *et al*., 2011). The epiRILs show variation in biomass productivity, especially when challenged with weed competitors and biotic stress, driven partly by complementarity among epigenotypes (Latzel *et al*., 2013). Variation in complex traits such as flowering time and root length is also influenced by epigenetic variation in segregating DNA methylation changes (Cortijo *et al*., 2014). In canola, recursive selection on epigenetic features of energy use efficiency showed higher yield potential and inheritance of acquired methylation patterns and agronomic characteristics (Hauben *et al*., 2009). These observations advance the hypothesis that induced epigenetic variation can be exploited effectively for selection in crop improvement.

*MutS HOMOLOG1* (*MSH1*) is a plant‐specific homolog of the bacterial DNA repair gene *MutS* (Abdelnoor *et al*., 2003). MSH1 is a nuclear‐encoded protein that is dual‐targeted to mitochondria and plastids, and depletion of MSH1 influences both mitochondrial and plastid properties (Xu *et al*., 2011). In *Arabidopsis msh1* T‐DNA insertion lines, phenotypes include leaf variegation, reduced growth rate, delayed flowering, extended juvenility, altered floral morphology, aerial rosettes and enhanced secondary growth (Xu *et al*., 2012). These mutants also show tolerance to heat, high light and drought stress (Shedge *et al*., 2010; Virdi *et al*., 2016; Xu *et al*., 2011). These pleiotropic phenotypes are largely attributed to depletion of MSH1 from plastids, evidenced by hemi‐complementation analysis (Xu *et al*., 2012), and the *msh1*‐triggered plastid changes condition genomewide methylome repatterning (Virdi *et al*., 2015). Similarly, detailed transcriptome analysis of *msh1* mutants reveals wide‐ranging changes in gene expression related to defence response, abiotic stress, MAPK cascade, circadian rhythm and phytohormone pathways (Shao *et al*., 2017).

RNAi suppression of *MSH1* in monocot and dicot species produces an identical range of developmental phenotypes (de la Rosa Santamaria *et al*., 2014; Xu *et al*., 2012; Yang *et al*., 2015). The altered phenotypes are somewhat attenuated but stable after segregation of the RNAi transgene, producing *msh1* ‘memory’. In sorghum, crossing *msh1* memory lines with isogenic wild type gives rise to enhanced vigour phenotypes that appear to respond to selection in small‐scale studies (de la Rosa Santamaria *et al*., 2014). In tomato, *MSH1‐*derived vigour phenotypes are heritable in glasshouse and field conditions, graft transmissible and obviated by treatment with 5‐azacytidine, further implicating DNA methylation in this phenomenon (Yang *et al*., 2015).

Soybean (*Glycine max* (L.) *Merr*.) is the most widely grown legume in the world, second only to grasses in economic importance. Synergistic interactions between advances in breeding and agronomic practices have steadily increased soybean yields in the past century (Rowntree *et al*., 2013). Further improvement will face challenges from climate instability and limited genetic diversity, which calls for the implementation of novel tools and methodologies to benefit soybean performance over a broad range of environments (Rincker *et al*., 2014). In this study, we used the well‐known soybean variety ‘Thorne’ (McBlain *et al*., 1993) to investigate amenability of the *MSH1* system in exploiting epigenetic breeding potential. Glasshouse and large‐scale multilocation field trials showed enhanced yield in selected F_2:4_ and F_2:5_ epi‐lines. We document tapering of *MSH1*‐derived vigour in these lines by F_2:6_ and show evidence of buffering effects in epi‐populations across environments, thus reducing epitype‐by‐environment interaction and possibly stabilizing yield across locations. Transcriptome studies of epi‐lines from F_2:4_, F_2:5_ and F_2:6_ generations revealed genes and pathways that participate in the *MSH1*‐derived enhanced growth and its waning by later generations.

## Results

### *MSH1* suppression in soybean induces a characteristic pleiotropic phenotype that persists after transgene segregation

**Figure 1:**
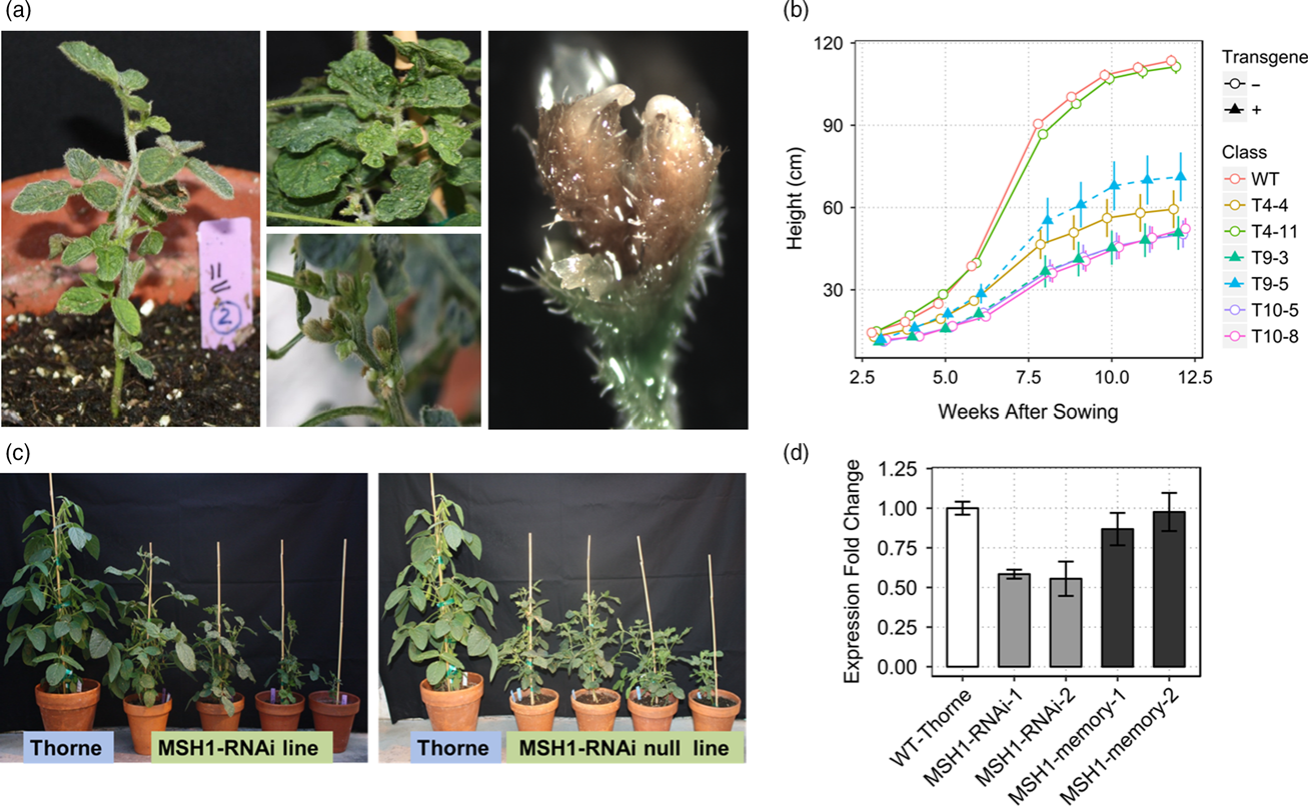
Characteristic phenotypes of *MSH1* suppression in soybean. (a) Characteristic *MSH1*‐RNAi phenotypes, dwarfing, wrinkled leaves, alterations of pod emergence and altered floral morphology showing flower with two stigmas. (b) Growth curve based on plant height in cm (measured weekly after 3 weeks of sowing) showing *MSH1*‐RNAi and transgene‐null *msh1* memory lines with reduced growth rate and higher variability within lines compared to wild type. (c) Similar range in plant height and leaf morphology variation from T7 *MSH1*‐RNAi (with transgene) and T10 *msh1* memory (without transgene) compared to wild type. (d) Gene expression profiling of T4 *MSH1*‐RNAi and T8 *msh1* memory lines for suppression of *MSH1* transcript level. Gene expression normalized to soybean actin levels and error bars represent SEM from three biological replicates.

RNAi suppression of *MSH1* in soybean produces phenotypic changes that include reduced growth rate, male sterility, enhanced branching and altered leaf and floral morphology (Figure 1a), similar to earlier reports in *Arabidopsis*, tomato and tobacco (Sandhu *et al*., 2007; Xu *et al*., 2011). Severely affected plants grow slower than wild type (Figure 1b) and show delayed flowering, extended juvenility and enhanced branching. The soybean *MSH1*‐RNAi T_0_ population did not produce visible variegation and/or male sterility, although 10%–20% of progeny from these lines (T_1_) showed wrinkled and puckered leaves. Almost 50% of the T_1_ plants were semi‐sterile, with increased flower drop and partially filled or empty seed pods. In subsequent generations, plants displayed a variable range of phenotypic severity.

Following transgene segregation, a proportion of progeny retained their acquired phenotypes of dwarfing, delayed flowering and altered leaf morphology for seven self‐pollinated generations tested to date (Figure 1c). The transgene‐null lines (T8 and T9), retaining altered phenotype while restored in *MSH1* transcript levels (Figure 1d), comprise the memory lines used in this study. Memory lines were classified based on their phenotype into intermediate (i) and extreme (e) designated i*MSH1* and e*MSH1,* respectively, while the remaining did not show any visible *MSH1* phenotype and were categorized n*MSH1* (Figure S1a).

### Transcript profiling of soybean *MSH1*‐RNAi lines shows correspondence of gene expression changes with phenotype severity

To evaluate the association of transcriptome changes with severity of *MSH1* phenotype, two soybean *MSH1*‐RNAi lines (transgene positive) differing in their phenotype severity were assayed by gene expression profiling with the Affymetrix Soybean Genome Array (GPL4592) (Xu *et al*., 2011). We used a stringent cut‐off (*P *< 0.05 and |log2(value)|>1) to call differentially expressed genes (DEGs) relative to wild‐type controls.

The severe phenotype plants showed differential expression of 2589 genes, whereas mild phenotype plants showed 154 DEGs, 114 of which were shared in common (Figure S2a). Both classes had far more up‐regulated genes, with severe showing 1656 up‐regulated and 933 down‐regulated genes and mild showing 145 up‐regulated and only nine down‐regulated genes (Figure S2b, Table S1). Gene Ontology (GO) analysis with AgriGO (Du *et al*., 2010) classified differences between the two phenotypic classes. While mild phenotype plants showed predominantly abiotic stress response, severe phenotype plants were more broadly affected in phytohormone, defence, immune and abiotic and biotic stress response pathways, reflecting a greater global stress response with increased phenotype severity (Figure S3a). A similar effect was seen in *Arabidopsis* (Shao *et al*., 2017), implicating a broader effect than would be conferred by organelle perturbation alone. Visualizing GO terms associated with enriched pathways using REVIGO (Supek *et al*., 2011), genes related to stress and calcium signalling were up‐regulated (Figure S2c), while photosynthesis and chromatin/cell cycle factors were down‐regulated, again reflecting global stress behaviour (Figure S2d, Table S2).

Cross‐species comparison of *MSH1*‐RNAi soybean transcriptome data with *Arabidopsis msh1* T‐DNA mutant (Shao *et al*., 2017) and tomato *MSH1*‐RNAi lines (Yang *et al*., 2015) showed that while individual genes did not necessarily overlap for differential expression between species, respective GO categories showed high coincidence (Table S3, Figure S3b). Defence, immune response, phytohormone, MAPKKK cascade and biotic and abiotic stress response categories were shared among the three species. Vitamin metabolism and senescence‐related genes comprised two categories that were enriched in the soybean *MSH1*‐RNAi line but not in tomato and *Arabidopsis*, reflecting a species‐specific response to the *msh1*‐associated perturbation. The results indicate that *MSH1* suppression confers strikingly similar changes in soybean, tomato and *Arabidopsis* in gene expression changes and associated phenotypes.

### Crossing soybean *msh1* memory lines to wild type produces epi‐lines with increased variation in adaptive traits

Recent studies have shown that crossing *msh1* memory lines to their isogenic wild‐type counterpart can influence growth vigour in *Arabidopsis*, sorghum and tomato (de la Rosa Santamaria *et al*., 2014; Virdi *et al*., 2015; Yang *et al*., 2015). To investigate the potential of *msh1*‐derived vigour in epi‐lines of soybean, assess inheritance and determine the longevity of enhanced growth behaviour through self‐pollination, we performed reciprocal crosses of *msh1* memory lines with wild‐type Thorne (Figure S4). Plants in the F_1_ generation were restored to the normal phenotype, ruling out cytoplasmic genetic changes for the *msh1* memory phenotype (de la Rosa Santamaria *et al*., 2014).

**Figure 2:**
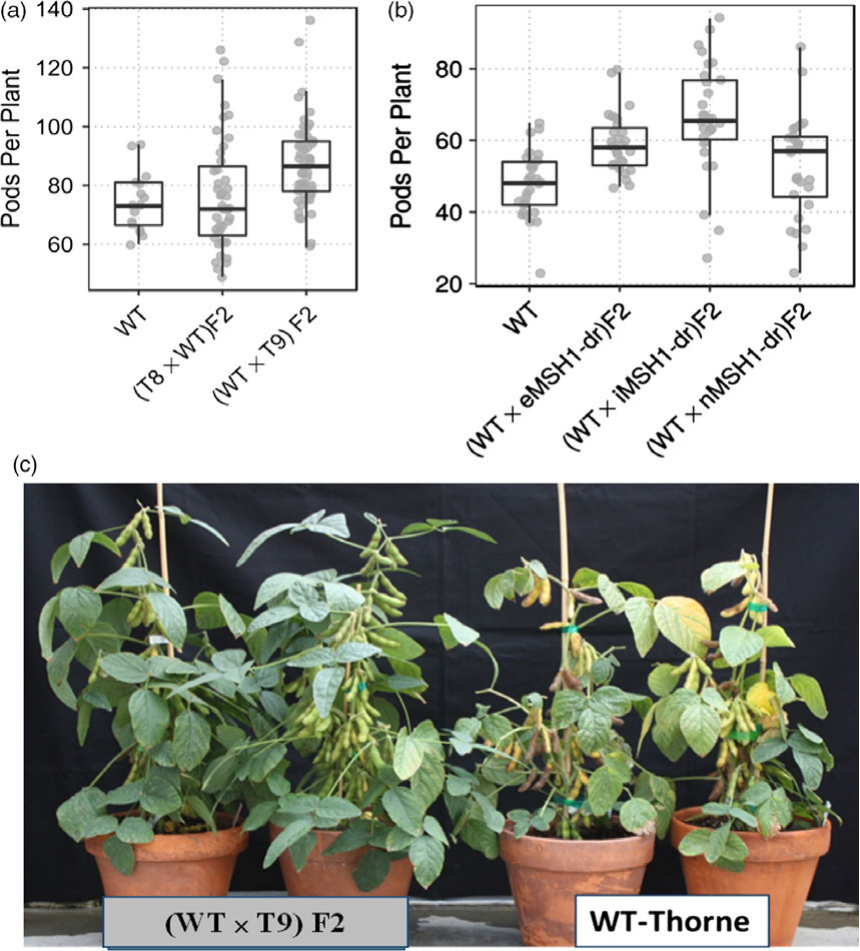
Increased variation for number of pods per plant in different epi‐F_2_ populations in the glasshouse. (a) Enhanced variation for pods per plant in two epi‐F_2_ populations compared to wild type grown under glasshouse conditions. (b) Variation in F_2_ performance for number of pods per plant in the glasshouse for populations derived from a range of *msh1* memory phenotypes (e*MSH* *1*, i*MSH* *1* and n*MSH* *1*). (c) WT × T9 epi‐F_2_ lines P37 and P34 showing increase in number of pods per plant and delayed maturity compared to wild type.

Derived epi‐F_2_ lines displayed a broader range of phenotypic variation than wild type for agronomic traits including number of pods (PP) and seeds per plant (SP), seed weight (SW), 100 seed weight (100SW), days to flowering (R1) and days to maturity (R8, Table S4). There was a significant difference in within‐genotype variance for number of pods per plant among wild type and the reciprocal F_2_ populations (Figure 2a, Bartlett test, *P =* 0.013). The variance estimate for wild type was 103.03, while for WT × T9 F_2_ and T8 × WT F_2_, it was 213.72 and 364.38, respectively. F_2_ populations also differed significantly in flowering time and maturity time, with a small proportion showing higher pod number per plant and delayed maturity (Table S4, Figure 2c).

We subsequently developed epi‐F_2_ populations by crossing wild type with three different phenotypic classes of nontransgenic memory lines, extreme, intermediate and normal phenotype (Figure S1a,b), as pollen donors. Similar to the previous reciprocal crosses, there were significant differences in variance between wild type and the three epi‐F_2_ populations for number of pods per plant (Figure 2b, Bartlett test, *P *= 0.0011). Increased variance for the measured traits was also observed among the three epi‐F_2_ populations. For example, epi‐F_2_ WT x eMSH1 showed lower variance than wild type for pods per plant and plant height, but higher variance for days to flowering (Table S5). Epi‐F_2_ WT x iMSH1 had a higher variance for pods per plant and days to flowering, while epi‐F_2_ WT x nMSH1 showed higher variance than wild type for all three measured traits (Table S5). These results suggest that *MSH1* epi‐populations represent different conditions, perhaps impacting the strategy for selection.

To investigate variation among derived epi‐lines and wild type under standard field conditions, we tested 30 F_2:4_ lines from each of the three populations, including 30 wild‐type sublines as shown in Figure S1b. These 120 lines were grown as random complete blocks (RCBD) in four Nebraska locations, Lincoln (SC), Clay Center (CC), Phillips (PH) and Mead (MD), with three replications per location for a total of 12 replications of two‐row, ten‐foot plots, with rows 3 m long and spaced 0.76 m apart. Data were collected on days to maturity, plant height, protein and oil concentration and total yield (Table S6).

Similar to glasshouse results for epi‐F_2_, we observed differences in variance components for total yield. Epi‐F_2:4_ nMSH1 showed ten times higher variance than wild type for total yield, while epi‐F_2:4_ eMSH1 showed variance similar to wild type (Table S7). We recorded single‐plant measurements for pod number per plant, number of branches and plant height, from ten randomly selected epi‐lines in each population along with ten wild‐type sublines from the multilocation field trial. Data were collected from five randomly selected plants from a plot, with two replicates in two locations, Mead and Clay Center. These two locations represent different agro‐ecological zones in Nebraska with contrasting soil types. From ANOVA tests, we saw no significant variation among strains or plants within strains for number of branches. For plant height, we saw significant variation among strains in F_2:4_ iMSH1 (*P* = 0.0096) and F_2:4_ nMSH1 (*P* = 0.0075). Epi‐F_2:4_ iMSH1 also showed significant variation among strains for pods per plant (*P* = 0.03), while wild type showed significant difference among plants within strains (*P* = 0.007, Table S8). These observations again indicate that epi‐lines may differ significantly in their *msh1* effects.

### Selected *MSH1* epi‐lines show increased yield compared to wild type in multi‐year field trials

To evaluate field performance of *MSH1* epi‐lines, F_2:4_ lines were derived from an upper 6% selection for number of pods per plant in the F_2_ generation. Thirty plants each from the selected F_2:3_ lines were grown in the glasshouse, and the upper half of these 30 plants, based on number of pods per plant, was bulked to form the ‘top 50% selection (S)’. In addition, equal numbers of seeds from all thirty plants were bulked to form the F_2:4_ ‘bulk composite’ (Figure S4). These F_2:4_ lines and wild type were grown as ten‐foot two‐row plots in the field at Havelock farm in Lincoln, Nebraska, during 2014.

**Figure 3:**
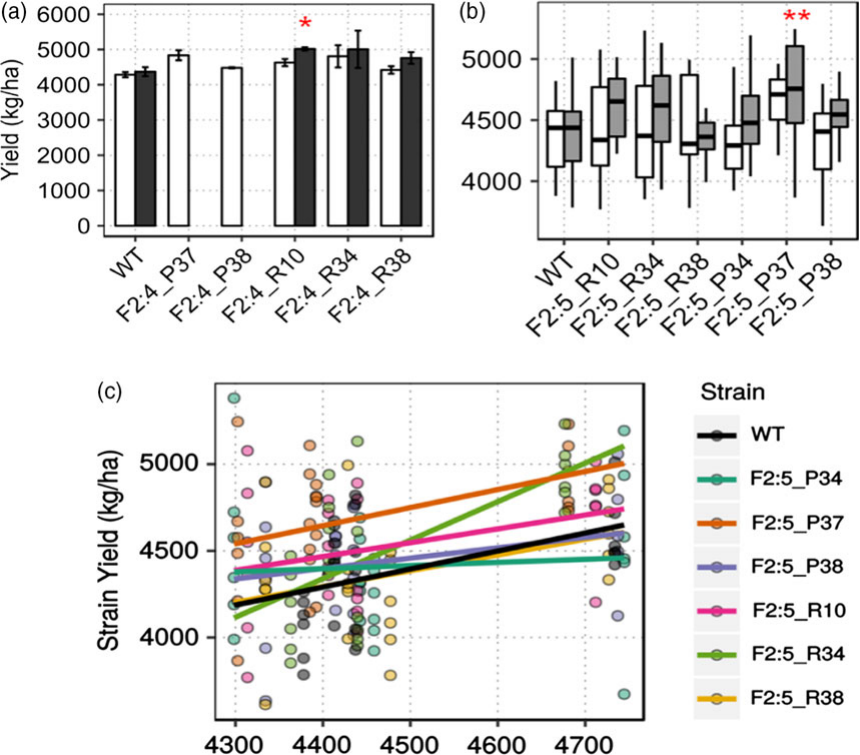
*MSH1‐*derived enhanced growth in field trials. (a) Enhanced growth measured as total seed weight in kg/ha normalized to 13% moisture for selected epi‐F_2:4_ lines in field experiments (*n* = 2). Asterisks represent statistical significance based on Welch's two‐sample *t*‐test (*P *= 0.02). Bar graphs in black represent ‘top 50% selection’ (S) lines (Figure S4) (b) Mean yield data from pooled locations showing enhanced yield in P37 F_2:5_ epi‐line compared to wild type (yield data pooled from three replicates each from four locations). Asterisks denote statistical significance based on *t*‐test (*P =* 0.00931). (c) Reaction norm plots showing superior yield performance of F_2:5_ P37 across environmental index for yield in kg/ha. Whisker plots in grey represent ‘top 50% selection’ (S) lines.

Wild‐type Thorne showed a mean yield of 4284.65 kg/ha, whereas bulk epi F_2:4_ line yields ranged from 4419.82 kg/ha to 4834.89 kg/ha and top 50% selection epi‐F_2:4_ line yields ranged from 4758.33 kg/ha to 5016.7 kg/ha. F_2:4_ R10S yielded significantly better than wild type (Welch's two‐sample *t*‐test, *P* = 0.02, Figure 3a) with a 95% confidence interval for yield gain between 283.3 and 1180.8 kg/ha. As a population, T8 × WT F_4_ yielded 4618.38 kg/ha and WT × T9 F_4_ yielded 4657.85 kg/ha compared to wild type, which yielded 4284.65 kg/ha.

Derived F_2:5_ epi‐lines (Figure S4) were grown in four different Nebraska locations in the summer of 2015, Lincoln (SC), Clay Center (CC), Phillips (PH) and Mead (MD), with three replications at each site. Mean yield data pooled across locations showed that grain yield for F_2:5_ P37 was significantly higher than wild type by 301.8 kg/ha (*t*‐test, *P =* 0.00931, Figure 3b), an increase of seven percentage. Except for F_2:5_ R38, all lines showed increased grain yield from 27 kg/ha to 301.8 kg/ha. Regression over an environmental index to visualize any epitype‐by‐environment (e × E) interactions showed F_2:5_ P34 to have a higher slope than wild type, but not significant by ANOVA. F_2:5_ P37 showed consistently higher yield than wild type across all environmental indices (Figure 3c).

To confirm that there was no penalty for enhanced seed yield in seed quality parameters, we measured seed protein concentration and oil concentration. There was no significant difference in seed protein concentration and 100 seed weight, but epi‐lines derived from T8 × WT crosses showed lower oil concentration compared to wild type. F_2:5_ lines from this population also showed earlier maturity compared to wild type (*P *= 0.0164). Lodging score did not show variation among the lines tested (Table S9).

F_2:6_ lines, developed from a glasshouse seed increase of 2014‐grown epi‐F_2:4_, showed no significant difference in mean yield compared to wild type (Figure S5), indicating that the enhanced growth effects taper back to wild‐type levels by F_6_. Consequently, these experiments demonstrate strongest yield enhancement at F_2:4_ and F_2:5_ generations, with the growth performance returning to wild‐type levels by F_2:6_, similar to the reported dissipation of epigenetic effects over generations in *ddm1* epiRILs (Cortijo *et al*., 2014; Roux *et al*., 2011).

### Progenies of wild‐type scion grafted on *MSH1*‐RNAi show increased yield in field trials

We tested whether enhanced growth could also be observed from *msh1*‐grafted progenies in soybean, drawing on previous reports in *Arabidopsis* and tomato (Virdi *et al*., 2015; Yang *et al*., 2015). For this experiment, we grafted three different phenotypic classes of *MSH1*‐RNAi rootstocks with wild‐type Thorne scions (Figure S6a), collected seeds from the graft plants and self‐pollinated them for one generation before planting in the 2015 multilocation field trial. Results showed significant yield increase in S2 grafted progenies over wild type (Figure S6b). The type of *MSH1*‐RNAi phenotype used as rootstock appeared to make a difference, with WT/n*MSH1*‐RNAi lines showing significantly higher yield compared to WT/WT graft (*t*‐test, *P =* 0.040) or WT (*t*‐test, *P =* 0.019), whereas the WT/i*MSH1* S2 line was marginally better than WT (*t*‐test, *P *= 0.052), and WT/e*MSH1* was not significantly different from wild type (Figure S6b). These results further support the nongenetic nature of enhanced growth and the involvement of mobile signals in the process.

### *MSH1*‐derived epi‐lines are more stable across environments

We performed ANOVA tests for interaction between strain and location within *MSH1* epi‐populations (Figure S1b). As expected, wild type showed strain × location interaction (*t*‐test, *P =* 0.0142), but the epi‐F_2:4_ populations showed no significant interaction (Table 1). To understand this outcome, we plotted the strain means across locations, showing more crossover interaction for wild‐type lines, particularly between SC and PH locations (Figure S7a). PH is a higher yielding site, with a mean yield of 4639.9 kg/ha, compared to SC, with a mean yield of 4403.04 kg/ha. Most lines from the epi‐population showed an increase in yield from SC to PH, while many of the wild‐type sublines declined. There was also a higher spread of values for wild‐type sublines at the MD location, which may be driving the interaction effects. Epi‐lines generally demonstrated higher yield consistency, with F_2:4_ i*MSH1* lines showing tighter grouping in both AMMI plots (Figure S7b) and reaction norms (Figure S7a) and performing well in good environments as shown by performance in PH.

**Table 1:** Test of significant epitype × environment interaction in wild‐type sublines and three different *MSH1* epi‐populations by ANOVA

	Df	Mean Sq	*F* value	Pr(>*F*)
Wild type				
Loc	3	5050936	17.27	<0.001
Rep(Loc)	8	292573	4.06	0.0002
Strain	29	116179	1.61	0.0295
Strain × Loc	87	104893	1.46	**0.0144** a
Residuals	230	72078		
WT × nMSH1 F_2:4_				
Loc	3	5209330	37.75	< 0.001
Rep(Loc)	8	138021	1.53	0.1479
Strain	29	205006	2.27	0.0004
Strain × Loc	87	89540	0.99	**0.5072**
Residuals	231	90262		
WT × iMSH1 F_2:4_				
Loc	3	5766440	22.98	<0.001
Rep(Loc)	8	250989	2.82	0.005
Strain	29	88107	0.99	0.483
Strain × Loc	87	85924	0.97	**0.564**
Residuals	229	88855		
WT × eMSH1 F_2:4_				
Loc	3	3380468	3.41	<0.1
Rep(Loc)	8	990356	10.66	1.18E‐12
Strain	29	112556	1.21	0.22
Strain × Loc	85	96811	1.04	**0.399**
Residuals	222	92910		

Different epi‐population (F_2:4_ eMSH1, F_2:4_ iMSH1 and F_2:4_ nMSH1) were developed from crossing wild‐type Thorne with *msh1* memory lines varying in phenotypic severity (Figure S1b). Table represents data from 30 sublines in each population grown as three replicates in four locations for a total of 12 replicates.

a Showing significant strain × location interaction in wild type.

Bold values represent data used to assess epi‐type x environment interaction.

From the variance component estimation, we see that wild type had more than eightfold higher G x E variance estimate than epi‐F_2:4_ populations for total yield (Table S7). There was no significant difference in G X E variance component for other traits such as maturity date, height and protein and oil concentrations. From the analysis of single‐plant measurements for among‐strain variation, wild type did not show any significant difference, while epi‐lines, particularly from F_2:4_ iMSH1, showed significant variation in plant height (*P = *0.0096) and number of pods per plant (*P =* 0.03), while F_2:4_ nMSH1 showed significant variation among strains for plant height (*P *= 0.007, Table S8). This inherent variation partly explains the buffering capacity for these epi‐lines in different environments, leading to reduced e × E interaction. These results imply that *MSH1*‐derived vigour and phenotypic plasticity can provide higher yield stability across different environments, although more extensive testing would be necessary to quantify this effect.

### Putative expression signatures in *MSH1*‐derived, enhanced growth epi‐lines

To investigate biological processes underlying the *MSH1*‐derived enhanced yield phenotypes in epi‐lines, we performed RNAseq analysis with the two epi‐lines R10 and P37 in F_2:4_, F_2:5_ and F_2:6_ generations and their respective wild‐type controls. These epi‐lines showed increased yield in F_2:4_ and F_2:5_ generations, while this enhancement diminished by F_2:6_. We utilized this gradual reversion phenomenon to identify signatures of enhanced growth and their change across generations.

To eliminate the possibility of seed contamination in the epi‐lines, we analysed the RNAseq data with the genome analysis toolkit (GATK) pipeline to identify SNPs from the alignment files. Plotting SNPs across the lines showed no haplotype blocks co‐segregating with the enhanced yield lines (Figure S8a,b), ruling out the possibility of seed contamination, and is consistent with our hypothesis of epigenetic regulation in *MSH1*‐derived epi‐lines in the absence of genetic changes.

**Figure 4:**
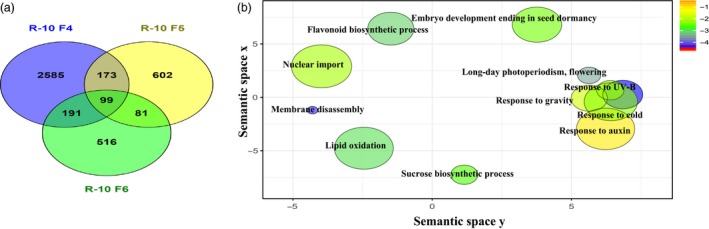
Gene expression changes and associated pathways in *msh1*‐derived epi‐lines with increased yield. (a) Venn diagram showing overlap of DEGs in enhanced growth epi‐line R10. (b) REVIGO plot showing non‐redundant GO terms associated with DEGs in epi‐line R10, enhanced growth epi‐F_2:4_ compared to epi‐F_2:6_, which showed yield similar to wild type. GO terms (*P *< 0.05) obtained from SoyBase were used in REVIGO tool from AgriGO with modified R script for plotting.

RNAseq results show R10 F_2:4_ with the greater mean yield gain, to display the highest number of DEGs compared to wild type, with 3048 DEGs, 1259 up‐regulated and 1789 down‐regulated. R10 F_2:5_ and R10 F_2:6_ showed 955 and 887 DEGs, respectively (Table S10, Figure 4a). We detected 682 DEGs in common between the two epi‐lines R10 F_2:4_ and P37 F_2:4_, accounting for 65% of DEGs in P37 F_2:4_ (Figure S9a). GO enrichment (SoyBase) and REVIGO analysis from these DEGs showed up‐regulation of stress response pathways (innate immune response, defence, abscisic acid signalling pathway) and down‐regulation of metabolism (protein phosphorylation, cellular response to phosphate and magnesium starvation, phosphate ion homeostasis and galactolipid biosynthesis) (Figure S9b). Several genes related to plastid function and development (Plastid organization, PS II assembly, bilateral symmetry, adaxial/abaxial pattern specificity, response to far‐red light and signal transduction) were differentially expressed only in R10 F_2:4_. As R10 F_2:4_ was derived from crosses with *msh1* memory line as female parent, these changes are likely remnants of the *msh1* memory effect.

To identify signature gene expression changes underlying the enhanced growth effect in epi‐F_2:4_ lines and the return to wild‐type levels by epi‐F_2:6_, we compared gene expression changes between F_2:4_ and F_2:6_ within the same lineage. To ensure direct comparison, we omitted genes that were differentially expressed in epi‐F_2:6_ vs WT_F6_ and WT_F4_ vs. WT_F6_ comparisons. This resulted in a filtered set of 545 DEGs in R10 and 454 DEGs in P37.

Auxin response genes were consistently modulated in both R10 and P37 epi‐lines. In R10 F_2:4_ vs F_2:6_ comparisons, we detected changes predominantly in sucrose biosynthesis as well as gravitropism and auxin stimulus response pathways (Figure 4b, Table 2), whereas in P37 F_2:4_ vs F_2:6_ comparisons, genes related to auxin response and protein phosphorylation were enriched (Table 2). A total of 40 DEGs (ca 8%) were common between the two epi‐lines. These genes represented auxin response, cell wall and cell cycle, and stress‐related genes (Table 3). The 40 genes were not necessarily modulated in the same direction in the two epi‐lines, perhaps emphasizing the role of circadian regulators in modulating the expression of these genes (Sanchez *et al*., 2018).

**Table 2:** Enriched GO terms associated with *MSH1*‐derived enhanced growth in R10 and P37 epi‐lines

Type	GO_id	GO_count	Expressed	Expected	*P_*adj	GO_desc
R10F4 vs. R10F6^a^	GO:0005986	35	6	0.5	0.0049	Sucrose biosynthetic process
	GO:0009629	23	5	0.3	0.0083	Response to gravity
	GO:0009733	1020	31	14.3	0.0408	Response to auxin stimulus
P37F4 vs. P37F6^b^	GO:0009733	1020	26	10.4	0.0127	Response to auxin stimulus
	GO:0006468	2386	7	24.4	0.038	Protein phosphorylation

a Represents DEGs between enhanced growth epi‐line R10 F_2:4_ (derived from epi‐population with *msh1* memory line as female parent) compared to R10 F_2:6_ line with yield similar to wild type.

b Represents DEGs between enhanced growth epi‐line P37 F_2:4_ (derived from epi‐population with *msh1* memory line as pollen donor) compared to P37 F_2:6_ line with yield similar to wild type.

**Table 3:**
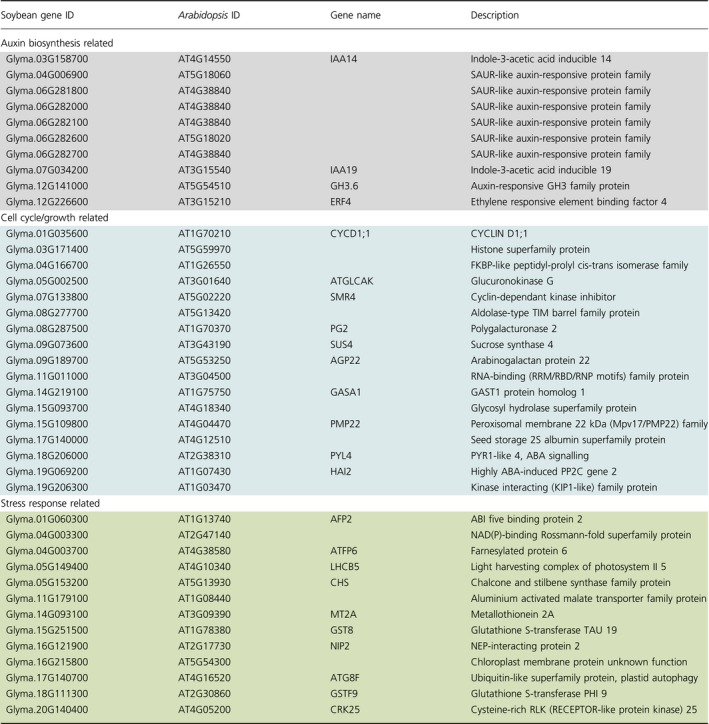
Common DEGs in two enhanced growth epi‐F_2:4_ lines, R10 and P37 compared to its respective epi‐F_2:6_

Auxin response genes include IAA19, a positive regulator of plant growth (Kohno *et al*., 2012) required for PIF4‐mediated modulation of auxin signalling (Sun *et al*., 2013). SMALL AUXIN UP RNAs (SAURs) were differentially expressed in both epi‐lines. SAUR genes are involved in cell expansion and development, particularly for integrating hormonal and environmental signals that regulate plant growth (Li *et al*., 2015; Ren and Gray, 2015). These data provide candidate pathways underpinning the growth behaviour in *MSH1* epi‐lines.

## Discussion

Previous studies have shown the influence of *MSH1* perturbation for altering growth in *Arabidopsis*, sorghum and tomato (de la Rosa Santamaria *et al*., 2014; Virdi *et al*., 2015; Yang *et al*., 2015). The present study exploits epigenetic variation induced by *MSH1* perturbation in soybean to develop epi‐lines that displayed an increase in seed yield from selected F_4_ and F_5_ families, subsiding by the F_6_ generation, under large‐scale field conditions. Epi‐lines showed reduced epitype‐by‐environment interaction, inferring contribution of the *MSH1* effect to buffering across environments. Gene expression profiling within the derived epi‐lines uncovered pathways modulated in the enhanced growth F_4_ and F_5_ cycles that returned to wild‐type levels by F_6_. Effects were particularly pronounced in auxin response pathways, suggesting their possible utility as candidate markers in early selection. Observation of auxin response pathway modulation in tomato epi‐lines further strengthens this argument (Yang *et al*., 2015).

Disruption of *MSH1* in both monocot and dicot plant species produces remarkably similar developmental reprogramming phenotypes that are independent of transgene segregation (Xu *et al*., 2012). Apart from conditioning a similar phenotypic response, *MSH1* disruption in various plant species show similar transcriptome behaviour, including changes in immune and defence, circadian rhythm, phytohormone and abiotic stress response pathways (Figure S3b). Methylome behaviour in *msh1* memory lines of *Arabidopsis* and tomato are also consistent (Sanchez *et al*., 2018), further reiterating cross‐species conservation for the *MSH1* effect.

In *Arabidopsis*, epigenome disruption through crossing wild‐type *Col‐0* with *met1*‐derived epiRILs triggers reprogramming of DNA methylation and changes in gene expression patterns in the F_1_ epi‐hybrids (Rigal *et al*., 2016). Similarly, crossing soybean *msh1* memory lines to isogenic wild type brings together two genetically identical genomes varying in DNA methylation patterns, creating conditions for widespread changes in DNA methylation and gene expression. The increased phenotypic variation in agronomic traits seen in F_2_ populations may be a consequence of segregation of these novel methylation patterns and chromatin changes.

Derived F_2:4_ epi‐lines showed significant variation for agronomically important traits like yield and days to maturity. Increasing variation in the population is considered beneficial under challenging conditions (Herman *et al*., 2014). Similar to bet hedging under different environments, epigenetically variable lines should be favoured, as a portion of the individuals are more suited to the prevailing environmental conditions, providing buffering capacity for the population (Herman *et al*., 2014). Our data, albeit early stage, support this notion by displaying reduced epitype‐by‐environment interaction than was observed in isogenic wild type across environments.

All six lines selected from top performing F_2_ plants showed a reduction in enhanced growth by F_2:6_, further confirming the epigenetic nature of *MSH1‐*derived growth changes, with similar dissipation patterns described previously in *Arabidopsis ddm1* epiRILs (Cortijo *et al*., 2014; Roux *et al*., 2011). A recent study has suggested that stability and switching of acquired epigenetic states are influenced by DNA sequence composition and repetitiveness (Catoni *et al*., 2017). It is also speculated that methylation variation not linked to a causal genetic variant tends to be less stable than when directly linked to genetic change (Schmitz *et al*., 2013). We deployed a strict top 6% selection in the F_2_ generation from each population for further evaluation. We assume that a more relaxed selection from these populations might show sustained enhanced growth for extended generations beyond F_2:6_.

GO enrichment analysis of DEGs in derived epi‐lines with increased yield showed changes in genes associated with photosynthesis, plastid organization, defence, immune response, light response and phytohormones. These pathways are also differentially modulated in *msh1* mutants (Shao *et al*., 2017), and similar gene expression changes in stress and phytohormone pathways are seen in *MSH1*‐derived epi‐F_3_ lines of tomato (Yang *et al*., 2015). Soybean epi‐line R10 F_2:4_ (*msh1* memory line as female parent) showed greater correspondence with the gene expression patterns of *msh1* mutants than did epi‐line P37 F_2:4_ (wild type as female parent). These observations suggest that the *msh1* mutant profile represents both organellar and epigenetic contributions to a global gene expression repatterning, and we are seeking to further dissect this phenomenon.

Immune and defence response genes were consistently up‐regulated in the two soybean epi‐lines, in contrast to their repression in *Arabidopsis* F_1_ plants from ecotype hybrids (Groszmann *et al*., 2015; Miller *et al*., 2015), perhaps reflecting a fundamental difference between *MSH1*‐derived enhanced growth and heterosis. Comparison of gene expression changes in F_2:4_ vs. F_2:6_ within a single lineage offers a unique system to understand the pathways associated with enhanced growth in the *MSH1* system. By this analysis, auxin response genes emerge in both epi‐lines tested to date, consistent with previous reports from *Arabidopsis* ecotype hybrids (Groszmann *et al*., 2015; Wang *et al*., 2017) and with previous studies in *MSH1‐*derived tomato epi‐F_3_ lines (Yang *et al*., 2015).

SAUR genes are implicated in regulating plant growth through sensing hormone and environmental cues (Li *et al*., 2015; Ren and Gray, 2015). These genes encode small proteins unique to plants that are found in tandem arrays or as segmental duplications of closely related genes (McClure and Guilfoyle, 1987) so that assigning a function to each SAUR gene is challenging. Recent evidence suggests an emerging relationship between phytohormones and epigenetic changes such as histone modification, chromatin remodelling and DNA methylation repatterning. Thus, coordinated changes in epigenomes may be one of the outcomes of plant hormone crosstalk (Yamamuro *et al*., 2016).

Sucrose biosynthetic pathway genes were also differentially expressed in R10 F_2:4_ relative to R10 F_2:6._ Starch metabolism changes in *Arabidopsis* serve as a means to enhance biomass and oil‐seed production while maintaining oil quality parameters (Liu *et al*., 2015). Sucrose synthase (SUS) enzymes play an important role in storage‐reserve accumulation in *Arabidopsis* (Fallahi *et al*., 2008), and similarly, fructokinases (FRKs) are important for storage‐reserve accumulation and embryo carbon catabolism (Stein *et al*., 2016). Whether these molecular signatures, both phytohormonal and metabolic, can be exploited in early generation selection to predict superior epi‐lines needs to be investigated further.

We provide evidence that novel epigenetic variation induced by *MSH1* suppression, following crossing and F_2_ segregation, can be inherited for at least three generations and bred for crop improvement with few rounds of selection to enhance and stabilize crop yield. It is unclear whether enhanced phenotypic plasticity will extend beyond this generational timeframe. This is a particularly intriguing question as relates to grafting, where no crossing is involved. These findings have interesting implications for plant breeding, epigenetics and transgenerational inheritance of nongenetic variation within plant genomes. The observed outcomes portend the utility of induced epigenetic variation within elite inbred lines, exploiting this variation to further enhance and stabilize agronomically important traits. One limitation of our study was that all the lines tested in the multilocation and multi‐year experiments were derived from only five different crosses and a similarly limited number of graft events, making it difficult to assess the frequency and effect of *msh1* memory and *MSH1* suppression phenotypes on crossing and large‐scale grafting outcomes. More work is now needed on molecular signatures of the ideal *msh1* suppression and memory lineages that will predict downstream performance and durability of the epigenetic effect.

## Materials and methods

### RNAi constructs and transformation

A 557‐bp segment encoding amino acids 945–1131, which represents the region following the ATPase domain (V) and spanning to the end of the GIY‐YIG homing endonuclease domain (VI) of the soybean *MSH1* gene, was PCR‐amplified using primers Soy‐MSF4 (5′‐ATCAGTTGGTTTATGCTAAGGAAATGCT‐3′) and Soy‐3Rbam (5′‐ TATGTATACAGGTTGGAAGTGCCAAAATTCCTATG‐3′). The PCR‐amplified fragment was cloned in forward and reverse orientation flanking the second intron of the *Arabidopsis* small nuclear riboprotein (At4g02840) in the pUCRNAi vector provided by Dr. H. Cerutti (University of Nebraska‐Lincoln) and later transferred into pPTN200 (pPZP family binary vector), which carries the *BAR* gene with nopaline synthase promoter and 3′UTR terminator. The hairpin sequences were placed under the control of 35S Cauliflower Mosaic Virus (CaMV) promoter with a duplicated enhancer and terminated by its 3′ UTR. The final vector CIPB‐7 was used to transform soybean by the cotyledonary node method of *Agrobacterium‐*mediated transformation (Xing *et al*., 2000; Zhang *et al*., 1999), and the herbicide Basta was used for selection of transformants.

### Plant material and growth conditions

For glasshouse studies, seeds were sown into moist peat pots containing standard potting mix and transferred to 8″ pots after 2 weeks. Plants were grown under 16‐h light/dark cycle at 28°C. Days‐to‐flowering (R1) was measured as number of days from sowing to one open flower at any node on the stem. Days‐to‐maturity (R8) was measured as number of days from sowing until 95% of pods were mature and brown. Plant height was taken at the R8 developmental stage as the distance between the soil surface and the apical meristem of the main stem. All plants were hand‐harvested individually, and number of pods was recorded before threshing to obtain number of seeds per plant. Near‐infrared (NIR) technology was used to determine protein concentration, oil concentration and moisture content of the seeds. Total seed weight was normalized to 13% moisture level.

Grafting was performed in the glasshouse on *MSH1*‐RNAi and *msh1* memory lines. Wild‐type seedlings at 12–14 days after sowing were used as scion and grafted onto 10‐day‐old rootstocks of wild‐type control or *MSH1* lines by the wedge grafting technique (Bezdicek *et al*., 1972; Kiihl *et al*., 1977) with necessary modifications. Seeds were collected from the grafted scion, and 30 plants from each graft were grown for one generation (S1) in the glasshouse and bulk harvested to obtain S2 seeds. Graft S2 lines were grown as two‐row, ten‐foot plots in multilocation field trials with three reps in each location for a total of 12 replications per graft.

During 2014 summer, twelve epi F_2:4_ lines with wild type were grown as four row plots (3 m long and 0.76 m apart). All data, including plot yield, were collected on the centre two rows of each plot. Emergent seedlings in each plot were counted 2 weeks after sowing to determine seed density, and four epi‐lines which had lower than 24 seeds per metre were discarded from further analysis. All lines were grown in a completely randomized design with two replicates. Rows were hand‐harvested and threshed on site, and grain yield measured as total seed weight for the plot adjusted to 13% moisture and converted to Kg/ha.

In 2015, a multilocation trial was conducted at four different Nebraska locations: Lincoln, Mead, Clay Center and Phillips. Lines were grown as two‐row plots (2.9 m long and 0.76 m apart) with 24–26 seeds per metre. In separate experiments, 12 F_2:5_ lines and six F_2:6_ lines from the reciprocal cross experiment were grown as random complete blocks (RCBD) with three replications at each location. In another experiment, 30 epi‐lines each from three epi‐F_2:4_ populations were grown along with 30 wild‐type sublines in RCBD with three replicates in four locations. Grain yield was measured as combined harvestable seed yield adjusted to 13% moisture. Height was recorded as the average length of the main stem from the soil surface to tip of the plant, expressed as the average of three individual plants in a uniform section of the row. Maturity date was recorded as number of days from planting until R8 stage, and lodging was scored from 1 to 5, with 1 indicating all plants in the plot erect, 3 indicating a plot average of plants at a 45‐degree angle and 5 showing all plants prostate on the ground. Single‐plant measurements were recorded from ten randomly selected lines in each population in two locations, Mead and Clay Center, with two replicates. In each plot, five randomly selected plants were marked and measurements were taken for pods per plant, number of branches, number of nodes and height.

### Phenotypic data analysis

For ANOVA of main effects and interactions in 2014 and 2015 field experiments, trait values were first fitted using the ‘lm’ function in R with the linear model *y*_*ijk*_ *˜ line*_*i*_ *+ env*_*j*_ + (line*env)_*ij*_ *+ (rep/env)*_*kj*_ *+ e*_*ijk*_, where *line*_*i*_ is the main effect of line *i*,* env*_*j*_ is the main effect of environment *j*, (line*env)_*ij*_ is the interaction between line *i* and environment *j*,* (rep/env)*_*kj*_ is the effect of replicate *k* nested within environment *j*, and *e*_*ijk*_ is the residual error; all independent variables were treated as fixed effects. Tests for significant effects and interactions were then performed using the ANOVA function within the ‘car’ R package. In the 2015 multilocation trial, outliers for grain yield were identified based on a threshold of more than 2× the interquartile range below the first quartile or above the third quartile (resulting in four observations removed).

For phenotypic analysis within and across multiple environments (the 2015 multilocation trial), mean trait values and corresponding confidence intervals were estimated for each line using the ‘lme4’ R package with the linear mixed model *y*_*ijk*_ *˜line*_*i*_ *+ env*_*j*_ + (line*env)_*ij*_ *+ (rep/env)*_*kj*_ *+ e*_*ijk*_, where *line*_*j*_ was treated as a fixed effect and *(rep/env)*_*kj*_ was treated as a random effect. Tests for significant differences in line means were performed using general linear hypothesis tests with the ‘multcomp’ R package, with *P*‐values adjusted using the Benjamini–Hochberg method. After fitting the model, variance components were extracted using ‘VarCorr()’ function in R. For analysis of single‐plant measurements in the field to look at strain variance and within line variance, data analysis was carried out using proc glm in SAS.

Joint regression analysis (Finlay and Wilkinson, 1963) was performed to assess individual line performance relative to the grand population performance across environments (i.e. environmental index). Trait data values for each line were regressed over the mean trait performance of all lines within that environment, excluding the line being estimated to avoid bias (Wright, 1976); the resulting slope of each line is an indicator of its response to environmental change compared to the population mean (Lynch and Walsh, 1998). AMMI plots were generated using the ‘agricolae’ R package.

### Microarray, RNAseq and SNP analysis

RNA preparation and processing for microarray assay have been described previously (Xu *et al*., 2011). We performed Gene Ontology (GO) analysis by converting the Affy probe ID into Soybean Genome ID (Phytozome) using a custom script in R. AgriGO (Du *et al*., 2010) analysis was performed on this list of differentially expressed genes. For comparative analysis, the best *Arabidopsis* BLAST hit for each differentially expressed orthologous gene in *MSH1*‐RNAi tomato (Yang *et al*., 2015) and severe *MSH1*‐RNAi soybean was used to generate GO enrichment and plotted as a heat map using custom R scripts.

For RNAseq, leaves from 4‐week‐old plants were harvested and frozen in liquid N2. Three biological replicates for each epi‐line, R10 and P37 from F_2:4_, F_2:5_ and F_2:6_ generations were sampled along with three generations of wild type (WT_F4_, WT_F5_ and WT_F6_). RNA was isolated with TRIzol (Invitrogen), followed by RNeasy (Qiagen) column purification. Sequencing was performed by BGI, generating 2 × 100 bp paired‐end reads with a mean of 25.6 million pairs per sample. After trimming bases below a quality score of 20, reads were aligned to the *Glycine max* reference obtained from Phytozome (cv. Williams 82, assembly v2.0) using STAR two‐pass method (Dobin *et al*., 2013) and allowing a mismatch rate of 0.04*(read length). This resulted in a mean unique mapping rate of 93.2%, or 97.3% when including multimapped reads. From STAR two‐pass alignment files, SNP detection was performed using the genome analysis toolkit (GATK) pipeline. SNP information from all samples was combined to create a total possible SNP list, filtered to only include SNPs supported by an alternate allele frequency of ≥0.75 and a read depth of ≥10. For every sample, if a SNP was not detected in a given position, it was assumed to be equal to the reference nucleotide. Only positions declared as SNPs in at least two of the 27 samples sequenced were retained as variable sites. Next, every sample was compared against the wild‐type samples of the other generations as the control, so that the wild‐type samples could also be evaluated; for example, WT_F4_, R10_F2:4_ and P37_F2:4_ were compared against WT_F5_ and WT_F6_. If a position had a different nucleotide than the wild‐type samples (only positions with an agreement among the wild‐type controls were considered), then it was considered a SNP relative to the wild‐type Thorne in our material.

All such SNPs were then plotted as depicted in Figure S8. Putative SNP haplo‐blocks did not co‐segregate with higher performance. For differentially expressed genes, reads were mapped to annotated genes (assembly 2, version 1, release 275) and then counted with strand‐specificity enforced. The Bioconductor package ‘sva’ was used to identify and remove a single surrogate variable related to sequencing lane batch effect. DESeq2 (Love *et al*., 2014) was used to normalize counts, estimate gene expression and identify differentially expressed genes (absolute log2 fold change ≥0.5 and a FDR < 0.05). SoyBase (http://soybase.org/) was used for GO enrichment analysis, and heat maps generated using custom R scripts.

## Conflict of interest

S. Mackenzie has served as cofounder for a startup company that is pursuing the MSH1 system for crop improvement.

## Author contributions

SM and SKKR designed the experiments and conceptualized the data, SKKR performed experiments, with YZX and AS in initial work on the transformants; SKKR, MSR, GG and RS analysed the data; SKKR wrote the original draft of the manuscript; SM and GG wrote, reviewed and edited the manuscript; and all authors read and approved the final manuscript.

## Supporting information


**Figure S1** Classification of *MSH1* memory phenotypes into extreme (e*MSH1*), intermediate (i*MSH1*) and normal phenotype (n*MSH1*).

**Figure S2** Gene expression changes and ReviGO terms associated with soybean severe *MSH1*‐RNAi lines.

**Figure S3** Transcriptome changes in soybean *MSH1*‐RNAi lines and cross species comparison of *MSH1*‐RNAi gene expression changes.

**Figure S4** Schematic representation of crossing scheme in *msh1* derived epigenetic breeding.

**Figure S5** Bar graph showing reduction in *MSH1*‐derived enhanced growth in epi F_2:6_.

**Figure S6** *MSH1*‐derived enhanced growth in S2 progenies of wild type scion grafted onto *MSH1*‐RNAi and *msh1* memory rootstock.

**Figure S7** Reaction norm and AMMI plots showing grouping of epi‐F_2:4_ and wild type sub‐lines across four different environments.

**Figure S8** Genetic distance profiles using SNPs from transcriptome data of wild type and epi‐lines.

**Figure S9** Overlap of genes and associated pathways in two epi‐lines R‐10 and P‐37 with enhanced growth.

**Table S1** Spreadsheet containing differentially expressed genes in mild and severe soybean *MSH1*‐RNAi.

**Table S2** Significant GO term enrichment from DEGs in mild and severe soybean *MSH1*‐RNAi.

**Table S3** Spreadsheet containing enriched GO terms for DEGs in soybean severe *MSH1*‐RNAi, tomato extreme *MSH1*‐RNAi, and *Arabidopsis MSH1* T‐DNA insertion mutant.

**Table S4** Summary of phenotypic data analysis for number of pods per plant, number of seeds per plant, seed weight, 100 seed weight, flowering time, and maturity time in reciprocal epi‐F_2_ population in the greenhouse.

**Table S5** Summary of phenotypic data analysis for number of pods per plant, days to flowering, and plant height in three epi‐F_2_ population compared to wild type in the greenhouse.

**Table S6** Summary of phenotypic data analysis for total yield, maturity, plant height, protein concentration, and oil concentration in wild type and epi‐F_2:4_ lines.

**Table S7** Summary of variance components for total yield in wild type and epi‐F2:4 populations.

**Table S8** Test of significant difference in variance for strain and plants within strains by ANOVA in wild type sub‐lines and three different epi‐F_2:4_ populations from single plant measurements of plant height, number of branches, and number of pods per plant.

**Table S9** Table containing summary of phenotypic data analysis for total yield, maturity date, plant height, protein, and oil concentration in wild type and enhanced growth epi‐F_2:5_ lines.

**Table S10** Spreadsheet containing DEGs in soybean *MSH1* epi‐lines R‐10 and P‐37 in F_2:4_, F_2:5_ and F_2:6_ generation compared to respective wild type control.
