# Supplementary figures and images for: An epigenetic breeding system in soybean for increased yield and stability
Source: Plant Biotechnol J. 2018 May 7;16(11):1836–47. doi: 10.1111/pbi.12919 (PMC6181216; doi:10.1111/pbi.12919)

a.

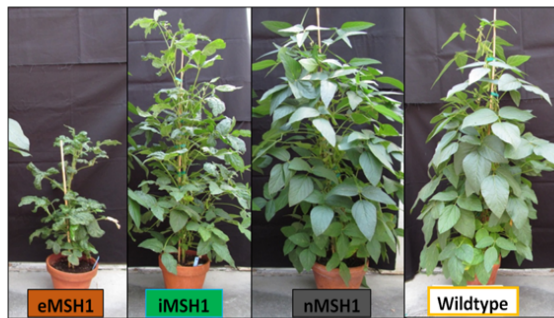

b.

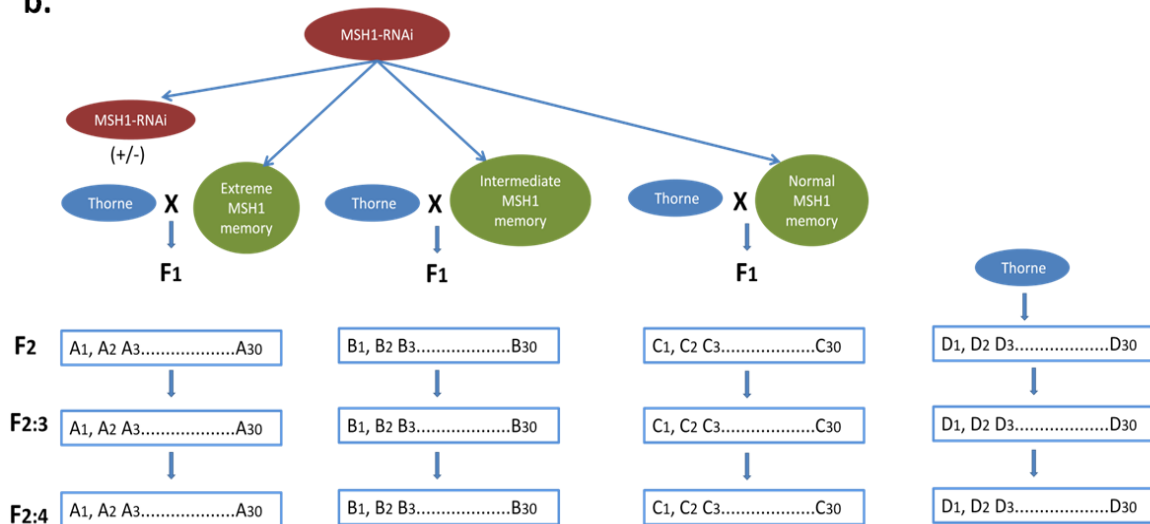

Supplement: Supplementary file 1 — Figure S1 Classification of MSH1 memory phenotypes into extreme (eMSH1), intermediate (iMSH1) and normal phenotype (nMSH1). [file PBI-16-1836-s001.pdf]

a.

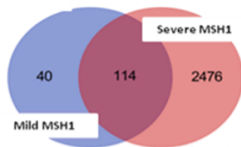

b.

|             | Up-regulated | Down-regulated |
|-------------|--------------|----------------|
| Severe MSH1 | 1656         | 933            |
| Mild MSH1   | 145          | 9              |

c.

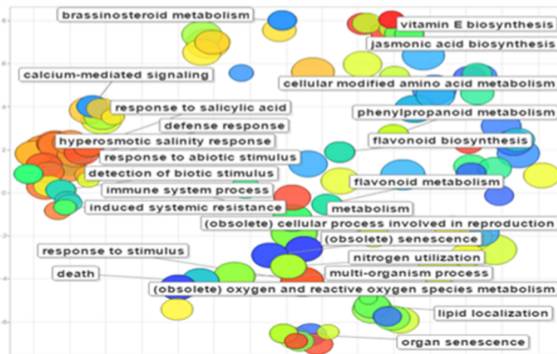

d.

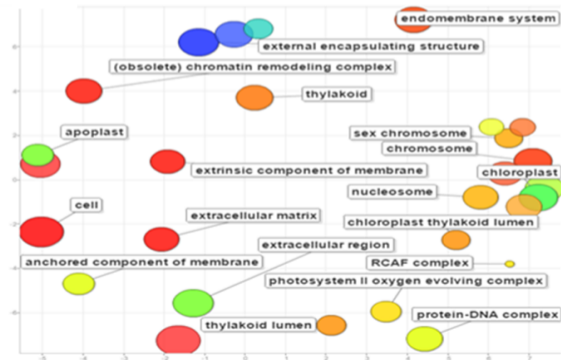

Supplement: Supplementary file 2 — Figure S2 Gene expression changes and ReviGO terms associated with soybean severe MSH1‐RNAi lines. [file PBI-16-1836-s017.pdf]

a.

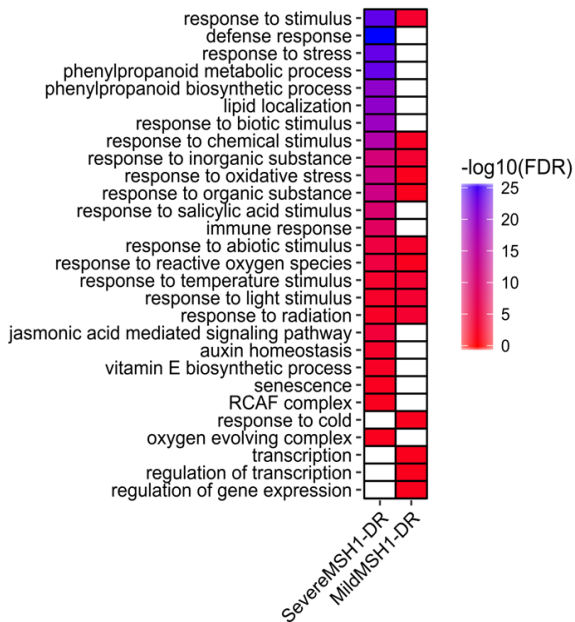

b.

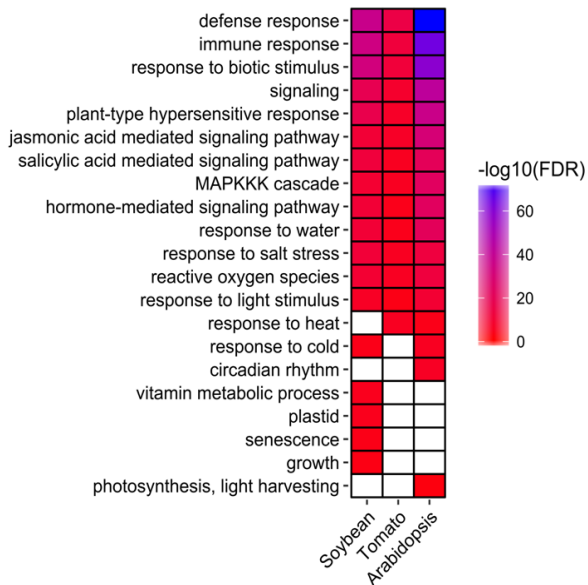

Supplement: Supplementary file 3 — Figure S3 Transcriptome changes in soybean MSH1‐RNAi lines and cross species comparison of MSH1‐RNAi gene expression changes. [file PBI-16-1836-s016.pdf]

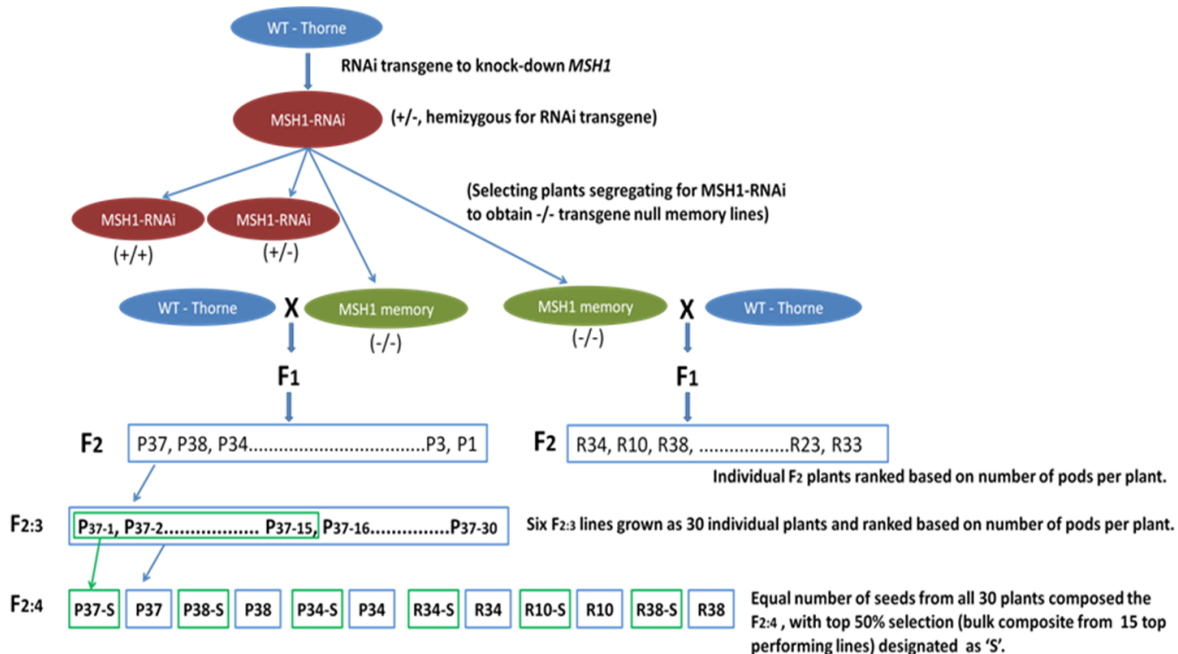

Supplement: Supplementary file 4 — Figure S4 Schematic representation of crossing scheme in msh1 derived epigenetic breeding. [file PBI-16-1836-s018.pdf]

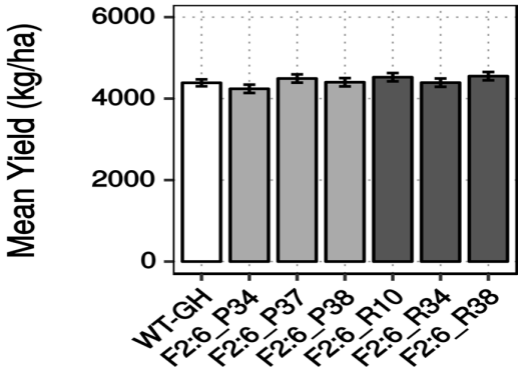

Supplement: Supplementary file 5 — Figure S5 Bar graph showing reduction in MSH1‐derived enhanced growth in epi F2:6. [file PBI-16-1836-s019.pdf]

a.

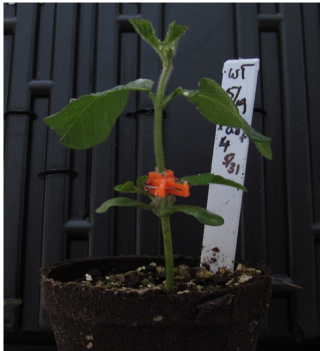

b.

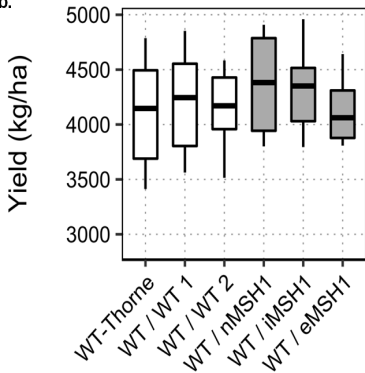

Supplement: Supplementary file 6 — Figure S6 MSH1‐derived enhanced growth in S2 progenies of wild type scion grafted onto MSH1‐RNAi and msh1 memory rootstock. [file PBI-16-1836-s002.pdf]

a.

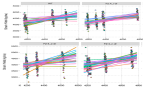

b.

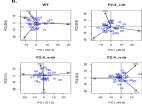

Supplement: Supplementary file 7 — Figure S7 Reaction norm and AMMI plots showing grouping of epi‐F2:4 and wild type sub‐lines across four different environments. [file PBI-16-1836-s003.pdf]

a.

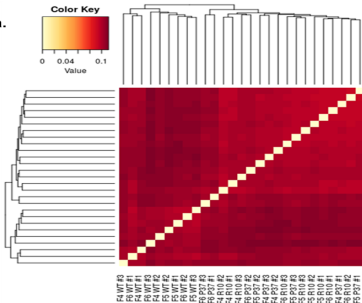

b.

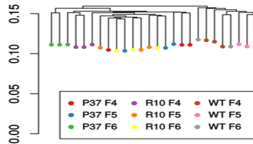

Supplement: Supplementary file 8 — Figure S8 Genetic distance profiles using SNPs from transcriptome data of wild type and epi‐lines. [file PBI-16-1836-s004.pdf]

a.

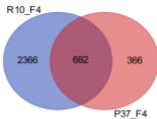

b.

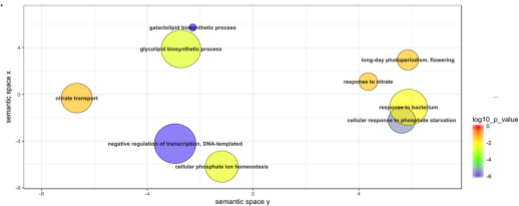

Supplement: Supplementary file 9 — Figure S9 Overlap of genes and associated pathways in two epi‐lines R‐10 and P‐37 with enhanced growth. [file PBI-16-1836-s005.pdf]
